# The impact of asthma education grounded in virtual reality technology upon the quality of life of pediatric patients with bronchial asthma

**DOI:** 10.3389/fped.2025.1588562

**Published:** 2025-06-05

**Authors:** Huijuan Wang, Qing Dong, Xiaowei Wang

**Affiliations:** ^1^ Pediatric Respiratory Department, The Sixth Hospital of Shijiazhuang, Shijiazhuang, Hebei, China; ^2^ Children's Rehabilitation Department, The Sixth Hospital of Shijiazhuang, Shijiazhuang, Hebei, China

**Keywords:** asthma education, bronchial asthma, asthma, asthma control, quality of life

## Abstract

**Objective:**

Asthma education stands as a pivotal element in the long - term management of asthma. This research endeavors to delve into the influence of virtual reality (VR) technology - based asthma education on the quality of life of children suffering from bronchial asthma.

**Methods:**

81 children with bronchial asthma from the Sixth Hospital of Shijiazhuang (Aug 2023–Aug 2024) were randomly divided into VR (40 cases) and asthma education (41 cases) groups. The latter received conventional education, while the former used VR technology. C - ACT, CDI, SCARED, MMAS - 8, and PAQLQ were used for assessment. Surveys on help - seeking times and VR device satisfaction were also conducted.

**Results:**

After and three months after the intervention, the VR group had higher C - ACT scores, more well - controlled asthma cases, lower CDI scores, fewer children with poor medication adherence, and higher MMAS - 8 scores than the asthma education group. Whether with severe or mild asthma, VR - based education improved C - ACT scores and the number of well - controlled cases. The VR group also had higher PAQLQ scores in symptoms, activity limitation, emotional function, and overall score. They accessed educational materials more often. 92.5% of parents/guardians were satisfied with VR devices and the program.

**Conclusion:**

Implementing VR technology - based asthma education for children with bronchial asthma can enhance the asthma control status, effectively mitigate the children's negative emotions, augment medication adherence, and ultimately elevate their quality of life.

## 1 Introduction

Bronchial asthma, a complex and heterogeneous disorder, stands among the most widespread respiratory afflictions besetting children across the globe (1). Statistical data disclose that around 14% of children worldwide contend with bronchial asthma, and more than one - third of adult patients with this condition initially developed it during their childhood (2). Despite the remarkable progress made in the treatment of bronchial asthma in recent years, only a small fraction of patients have managed to attain effective control over their symptoms (3). This scenario is especially prevalent among children. Poorly - controlled asthma not only aggravates the condition of young patients but also imposes a hefty burden on the affected children and their families, significantly impairing the daily lives, academic achievements, work productivity, and overall life quality of the children and their parents (4, 5). Research suggests that the life quality of children with bronchial asthma deteriorates as the control of their condition becomes less proficient (6).

The decline in the life quality of children with inadequately - controlled bronchial asthma can be ascribed to a plethora of factors, among which negative emotions such as anxiety and depression play a crucial role (7, 8). Studies have discovered that children with bronchial asthma are more than twice as likely to experience symptoms of anxiety and depression compared to their healthy counterparts (9). This is believed to be associated with a higher incidence of asthma attacks, more frequent medical consultations, and a lower life quality (10, 11). On one hand, owing to the distinctive psychological development characteristics of children, bronchial asthma itself can exert a profound and adverse influence on the psychological development of young patients, rendering them more susceptible to negative emotions such as anxiety and depression, which in turn affect their life quality (12, 13). On the other hand, the emergence of negative emotions can also diminish the compliance of children with asthma to their prescribed medications, resulting in suboptimal disease control, more frequent symptom exacerbations, and a more severe impact on their life quality (14).

Asthma education is regarded as an efficacious strategy for enabling children with bronchial asthma to achieve better control of their condition (15). As a vital component of non - pharmacological treatment for bronchial asthma, it is an intervention designed to assist patients with asthma in self - managing their condition and related burdens. It can enhance patients' asthma control by equipping them with disease - related knowledge and skills (16). Leading global respiratory, asthma, and health - related professional organizations advocate for the provision of education to patients with asthma as an integral part of comprehensive asthma management (17). Moreover, asthma education also contributes to enhancing the life quality of children with bronchial asthma (18). Nevertheless, in practical application, approximately 20%–50% of children with bronchial asthma fail to participate in and receive effective asthma education, which severely impedes efforts to improve their life quality (19). The primary cause of this phenomenon is that traditional classroom - based asthma education is often overly dull and unappealing for children, failing to capture their attention and interest effectively, thus significantly reducing the intervention's impact on young patients (20).

In recent years, with the advent of various novel technologies, virtual reality (VR) technology has found extensive applications in the fields of medicine and nursing (21). Interventions utilizing VR technology have proven to be highly effective in improving patients' symptom control and life quality (22). When patients engage with VR technology, they can relish immersive experiences and simulations. Additionally, VR technology can offer encouragement and motivation during the intervention process, enhancing patients' learning experiences (23). Furthermore, the immersive experiences and simulations provided by VR technology occur in a safe and agreeable environment, which is highly alluring to children and can effectively arouse their interest in the intervention (24). Therefore, this study aims to explore the impact of VR - based asthma education on the life quality of children with bronchial asthma through an intervention, with the hope of providing a reference for improving the current state of asthma education for these children and promoting their healthy development.

## 2 Methods

### 2.1 Patients

This research selected pediatric patients with bronchial asthma who presented at the Sixth Hospital of Shijiazhuang from August 2023 to August 2024 as the study subjects. The children were numbered according to their order of consultation, and the random number table method was utilized to evenly assign all the children to the VR group and the asthma education group. Meanwhile, according to the severity of the children's asthma, they were further divided into two subgroups: mild asthma and severe asthma (25).

The inclusion criteria for this study were as follows: (1) aged between 6 and 14 years; (2) diagnosed in accordance with the diagnostic criteria for bronchial asthma (17); (3) in a stable condition, in the clinical remission phase of asthma, and in a physical state conducive to collaborating to complete this research; (4) the asthmatic children and their parents possessing a certain degree of cognitive comprehension and communication capabilities; (5) being free from severe acute or chronic physical ailments, cognitive or mental impairments.

The exclusion criteria for this study were as follows: (1) asthmatic children accompanied by other systemic diseases, severe complications, or in an acute attack; (2) asthmatic children and their parents who had participated in other asthma health - education initiatives; (3) those with cognitive or mental disorders; (4) Children with attention deficit or those unable to concentrate.

This study was sanctioned by the Ethics Review Committee of the Sixth Hospital of Shijiazhuang, and informed consent forms were procured from all the children and their guardians.

### 2.2 Intervention approaches

All the children received standardized asthma drug treatment in accordance with the GINA guidelines, including inhaled corticosteroids (ICS) and short - acting β - agonists (SABA) (26). The types, doses, and administration frequencies of the medications were the same between the two groups. The medications were prescribed by their attending physicians, and the attending physicians also confirmed that the children responded effectively to the corresponding drug treatments. On this foundation, corresponding nursing intervention measures were carried out.

#### 2.2.1 The asthma education group

Implement regular asthma education interventions for the children in the asthma education group. Specifically: Upon the children's admission to the hospital, apprise the children and their parents of the relevant etiologies, manifestations, and influencing factors of asthma, and elucidate the precautions regarding medication to the children and their families. Ensure that the ward is well - ventilated, tranquil, immaculate, and cozy. Medical staff should meticulously monitor the children's conditions, encourage the children to boldly convey their genuine emotions, and relieve the psychological strain stemming from the children's misapprehensions about the disease. Offer guidance on environmental hygiene to the children and their families, instruct and inform them to steer clear of allergens to prevent the recurrence of asthma. Guide the children to partake in appropriate physical activities and uphold a salubrious diet. After the children are discharged from the hospital, conduct regular follow - ups with the children and their families to comprehend the frequency and control status of the children's asthma attacks, and provide comprehensive guidance to the children and their families to aid the children in managing bronchial asthma.

#### 2.2.2 The VR group

For the children in the VR group, asthma education grounded in VR technology was imparted, specifically as follows:

(1) Formation of an Intervention Nursing Team: An intervention nursing team was assembled, with the researcher designating a team leader. The team members included a pediatric attending physician boasting rich clinical experience, a clinical pharmacist, and four ward nurses.
(2) Creation of VR Health - Education Videos and Images: A panoramic camera was employed to capture 360° viewable VR videos and panoramic pictures. The content of the VR videos encompassed relevant domains such as disease - onset triggers, treatment modalities, medication regimens, daily care, dietary and exercise guidelines. The filming location for the videos and pictures was the children's ward. Two nurses respectively assumed the roles of the nurse and the child in the video. During the filming, the video was shot from the first - person vantage point of the child, with the panoramic camera positioned at the simulated child's head. The video portrayed the entire journey from the child's illness onset to hospitalization, specifically featuring medical staff apprising the child and parents of the disease - onset factors, intervention approaches, medication processes, and daily dietary and exercise guidance. The ward panoramic pictures and panoramic videos could only be viewed in 360° when played via a VR device.
(3) Development of VR Rehabilitation Training Software: The development of VR rehabilitation training software primarily entailed two stages. The first stage involved using a panoramic sports camera to film videos of rehabilitation function training. The second stage was to design the filmed video materials into a rehabilitation training software and import it into the VR device. The filming location for the software video materials was in the rehabilitation ward of our hospital, with one nurse impersonating the child. The filming was conducted from two perspectives: the first - person and the third - person perspectives of the child. When filming from the first - person perspective, the child was first requested to place the camera on the head, aligning the lens with the line of sight, and then perform pulmonary function training. When filming from the third - person perspective, the camera was first fixed on a support beside the child, and then the simulated child carried out pulmonary function training. The content of pulmonary rehabilitation function training included the utilization of a breathing trainer (training inspiratory muscle strength with a three - ball breathing trainer with resistance), breathing exercises (abdominal breathing and pursed - lip breathing), upper - limb muscle strength and endurance and accessory respiratory muscle training (weight - bearing chest - expanding exercises combined with breathing and weight - bearing double - hand raising exercises combined with breathing), and aerobic activities (jogging, skipping rope, etc.). The training was to be sustained for approximately 30 min daily, accompanied by elucidations and words of encouragement, and timely feedback was to be provided. After the filmed video materials were fashioned into a rehabilitation training software and imported into the VR device, the nurse could select corresponding functional exercise actions in the software according to the child's individual condition and set the exercise duration.
(4) Intervention via VR - Based Asthma Education: Upon the child's admission to the hospital, the nurse assisted the child in donning the VR device. The disease knowledge - education VR videos were played through the built - in virtual cinema software of the VR device. The child was required to wear the VR device for rehabilitation function training. If the condition allowed, the child could wear the VR device to engage in relevant functional training through the built - in mini - games, such as VR games like climbing and swimming, to enhance the child's upper - limb mobility and pulmonary respiratory function. VR health education was implemented throughout the child's hospitalization period, and the viewing schedule of the videos was arranged. If the child's condition altered and the child could not endure the education, the education content would be adjusted in accordance with the specific circumstances. The nurse would sign off after implementation to guarantee that each item was carried out. If the child experienced dizziness, nausea, or other discomforts during the playback, the playback would be paused. After the playback was completed, oral education would be provided to the child and parents.

After the child's discharge from the hospital, the child was instructed to regularly utilize the VR device at home to receive asthma education. The child's situation regarding symptom control, device usage, medication, etc., was to be promptly ascertained through various means such as the Internet and telephone. The problems confronted by the child after discharge were to be promptly addressed to solidify the effect of asthma education intervention.

All the children and their families underwent a two - month - long asthma education intervention.

### 2.3 Observation indicators

#### 2.3.1 Asthma control

Immediately after the asthma education program concluded and three months following its completion, the Childhood Asthma Control Test (C - ACT) questionnaire was utilized to assess and tabulate the children's asthma control status. The C - ACT questionnaire consists of seven items. It incorporates three inquiries for children to convey their overall viewpoints on asthma control, physical activity restrictions, coughing, and nocturnal awakenings, as well as three inquiries for caregivers to recollect the symptoms over the past four weeks, including daytime symptoms, daytime wheezing, and nocturnal awakenings. Medical personnel elucidated the questionnaire and assisted the children and their families in completing it. The initial four items were filled out by the children, and the final three by the families. The total score amounts to 27 points. A higher score signifies superior disease control. Specifically, a score exceeding 25 points implies excellent control, a score ranging from 20 to 24 points indicates basic control, and a score below 20 points suggests a lack of control (27).

#### 2.3.2 Negative emotions

Prior to and subsequent to the children receiving asthma education, the Screen for Child Anxiety Related Emotional Disorders (SCARED) and the Children's Depression Inventory (CDI) were employed for evaluation. The SCARED scale is applicable to children and adolescents aged 7–16 years. It comprises 41 items, with each item scored on a 0–2 scale. The total score ranges from 0 to 82 points. A higher score denotes more conspicuous anxiety in the children. The CDI scale is suitable for children aged 6–13 years, containing 27 items. Each item is scored on a 0–2 scale, and the total score ranges from 0 to 54 points. A higher score indicates more pronounced depressive emotions in the children (28).

#### 2.3.3 Medication adherence

Before and after the children underwent asthma education, the Morisky Medication Adherence Scale (MMAS - 8) was employed to statistically score the children's treatment adherence. The MMAS - 8 scale consists of eight questions. For questions 1–7, the alternative responses are “Yes” and “No”, scored 0 and 1 point respectively. For question 5, “Yes” is scored 1 point and “No” is scored 0 point. For question 8, the responses “Never”, “Occasionally”, “Sometimes”, “Often”, and “All the time” are scored 1, 0.75, 0.5, 0.25, and 0 points respectively. The full score is 8 points. A score below 6 points indicates poor adherence, 6–7 points indicates moderate adherence, and 8 points indicates good adherence (29).

#### 2.3.4 Quality of life

Before the asthma education program, immediately after its completion, and three months after the program ended, the Pediatric Asthma Quality of Life Questionnaire (PAQLQ) was used to statistically score the children's quality of life. The PAQLQ scale has three dimensions with 23 items, encompassing the three principal domains for evaluating quality of life: activity limitations, symptoms, and emotional function. The scoring system is on a 7 - point scale, with a minimum of 1 point and a maximum of 7 points. The sum of the scores of each item is the total score, with the total score ranging from 7 to 161 points. A higher score indicates a better quality of life (30).

#### 2.3.5 Number of times seeking help

During the period when the children received asthma education, the number of times the children or their family members in the VR group accessed the educational materials was recorded. Meanwhile, the number of times the children or their family members in the asthma education group sought additional help via forms such as email or phone calls was also statistically analyzed.

#### 2.3.6 Satisfaction with VR

After the intervention was completed, interviews were conducted with the children's family members to investigate their satisfaction with the VR device and the asthma education based on the VR device. They were asked whether they were satisfied with the VR device and the associated asthma education, and also about the problems they perceived.

### 2.4 Statistical methods

In this study, SPSS statistical software version 25.0 was employed to analyze all the data. Quantitative data were computed as mean ± standard deviation (x̅ ± sd). The independent - samples *t*-test was utilized for comparisons between groups, and the paired *t*-test was used for pre - and post - comparisons within the same group. Analysis of variance (ANOVA) was utilized for comparisons among multiple groups. Qualitative data were presented as numbers/percentages (*n*/%). The chi - square test was used for comparisons. When the *P*-value was less than 0.05, the difference was regarded as statistically significant.

## 3 Results

### 3.1 Fundamental information of the pediatric patients

Statistically speaking, this study enrolled 106 pediatric patients diagnosed with bronchial asthma who sought medical attention at the Sixth Hospital of Shijiazhuang from August 2023 to August 2024. Among them, the guardians of 15 pediatric patients (14.15%) declined their participation, and 7 patients (6.60%) met the exclusion criteria. Eventually, 84 patients were selected and randomly allocated into two groups: 42 pediatric patients in the VR Group and 42 pediatric patients in the Asthma Education Group. During the follow - up period, 2 patients in the VR Group were lost to follow - up, and 1 patient in the Asthma Education Group was lost to follow - up. In total, 81 patients completed the experiment, with 40 in the VR Group and 41 in the Asthma Education Group.

**Figure 1:**
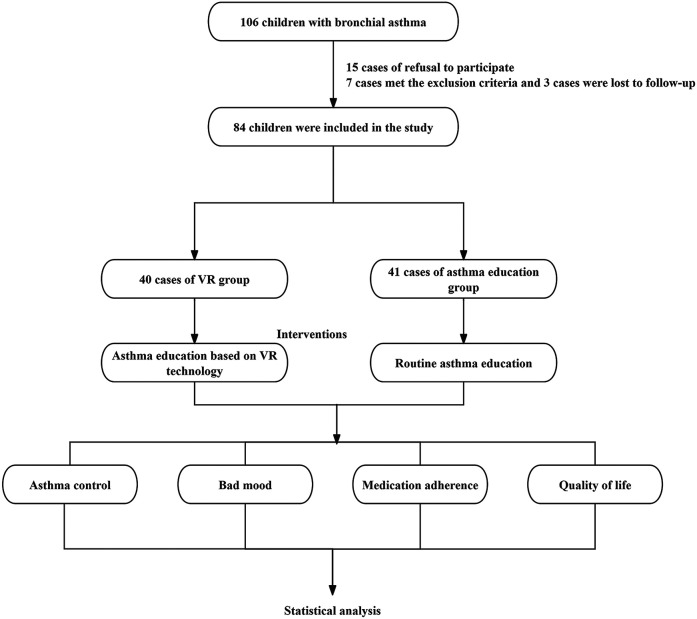
The flowchart of the experiment.

Statistically, within the VR Group, there were 22 male pediatric patients and 18 female pediatric patients, with a mean age of (8.50 ± 1.22) years, a mean disease duration of (21.20 ± 2.04) months, and a mean body mass index of (20.37 ± 0.88) kg/m^2^, Twelve pediatric patients had prenatal risk factors (such as maternal smoking during pregnancy, premature birth, and family history of asthma), 23 patients currently had risk factors or factors exacerbating asthma (including allergen exposure, second - hand smoke, air pollution, and pet - keeping), and 5 patients suffered from severe asthma. Among the pediatric patients in the Asthma Education Group, there were 21 male pediatric patients and 20 female pediatric patients, with a mean age of (8.54 ± 1.21) years, a mean disease duration of (20.68 ± 2.01) months, and a mean body mass index of (20.54 ± 0.83) kg/m^2^, Fifteen pediatric patients had prenatal risk factors (like maternal smoking during pregnancy, premature birth, and family history of asthma), 24 patients currently had risk factors or factors exacerbating asthma (such as allergen exposure, second - hand smoke, air pollution, and pet - keeping), and 3 patients had severe asthma. Analysis indicated that there were no statistically significant disparities in gender, age, disease duration, and body mass index between the pediatric patients in the VR Group and those in the Asthma Education Group (gender: *P* = 0.733; age: *P* = 0.892; disease duration: *P* = 0.254; body mass index: *P* = 0.371; prenatal risk factors: *P* = 0.530; current risk factors or factors exacerbating asthma: *P* = 0.925; number of patients with severe asthma: *P* = 0.682) (Table 1, Figure 1).

**Table 1:** Fundamental information of the pediatric patients.

Parameter	The VR group (*n* = 40)	The asthma education group (*n* = 41)	*t*/X^2^	*P*
Gender/*n* (male/female)	22/18	21/20	0.116	0.733
Age (years)	8.50 ± 1.22	8.54 ± 1.21	0.136	0.892
Duration of illness (months)	21.20 ± 2.04	20.68 ± 2.01	1.150	0.254
Body mass index (kg/m^2^)	20.37 ± 0.88	20.54 ± 0.83	0.900	0.371
Number of patients with prenatal risk factors	12	15	0.395	0.530
Number of patients with current risk factors or asthma exacerbating factors	23	24	0.009	0.925
Children with severe asthma	5	3	0.168	0.682

### 3.2 Asthmatic control efficacy in children

The outcomes of the C - ACT scoring system revealed that upon the conclusion of the intervention, in the VR cohort, the children's scores on the C - ACT questionnaire averaged at (23.65 ± 2.03). Specifically, 38 children in this group achieved asthmatic control, whereas 2 did not. In the asthma education cohort, the children's average C - ACT questionnaire score was (20.29 ± 2.50). Among them, 26 children had their asthma under control, while 15 did not. The C - ACT scores of the children in the VR cohort were significantly higher than those in the asthma education cohort (*P* < 0.001), and the number of children with controlled asthma in the VR cohort exceeded that in the asthma education cohort (*P* = 0.001).

Three months after the end of the intervention, the C - ACT questionnaire score of children in the VR group was (23.65 ± 2.03), and the asthma conditions of all children were under control. After comparison, the C - ACT questionnaire score of children in the VR group increased compared with that at the end of the intervention (*P* = 0.018), but there was no statistical difference in the number of children with controlled asthma compared with that at the end of the intervention (*P* = 0.474). The C - ACT questionnaire score of children in the asthma education group was (20.29 ± 2.50). Among them, 34 children had their asthma under control, while 7 did not. After comparison, there was no statistical difference in the C - ACT questionnaire score of children in the asthma education group compared with that at the end of the intervention (*P* = 0.068), but the number of children with controlled asthma in this group increased compared with that at the end of the intervention (*P* = 0.047). Three months after the end of the intervention, both the C - ACT questionnaire score and the number of children with controlled asthma in the VR group were higher than those in the asthma education group (C - ACT questionnaire score: *P* < 0.001; number of children with controlled asthma: *P* = 0.020) (Table 2).

**Table 2:** Asthmatic control efficacy in children.

Parameter	The VR group (*n* = 40)	The asthma education group (*n* = 41)	*t*/X^2^	*P*
C-ACT score after intervention	23.65 ± 2.03	20.29 ± 2.50	6.619	<0.001
Number of children with asthma under control	38	26	12.181	0.001
C-ACT score at 3 months after the completion of asthma education	24.60 ± 1.45*	21.17 ± 1.72	9.713	<0.001
Number of children with controlled asthma at 3 months after the completion of asthma education	40	34*	5.469	0.020

C-ACT, childhood asthma control test questionnaire.

* Compared with the same group after intervention, *P* < 0.05.

We analyzed the asthma control effects of asthmatic children with different asthma severities receiving different asthma education. The C - ACT questionnaire score of children with severe asthma who received VR - based asthma education was (20.20 ± 1.10), while that of children who only received conventional asthma education was (16.33 ± 0.58). Three months after the end of asthma education, the C - ACT questionnaire score of children with severe asthma who received VR - based asthma education was (21.80 ± 1.30), while that of children who only received conventional asthma education was (18.00 ± 1.00). There was no statistical difference in the C - ACT questionnaire scores of children with severe asthma in the two groups at three months after the end of asthma education compared with those at the end of asthma education (VR group: *P* = 0.069; asthma education group: *P* = 0.067). However, both at the end of the intervention and three months after the end of the intervention, the C - ACT questionnaire scores of children with severe asthma in the VR group were higher than those in the asthma education group (after the end of the intervention: *P* = 0.001; three months after the end of the intervention: *P* = 0.005). The C - ACT questionnaire score of children with mild asthma who received VR - based asthma education was (24.14 ± 1.61), while that of children who only received conventional asthma education was (20.61 ± 2.32). Three months after the end of asthma education, the C - ACT questionnaire score of children with mild asthma who received VR - based asthma education was (24.14 ± 1.61), while that of children who only received conventional asthma education was (21.42 ± 1.50). There was no statistical difference in the C - ACT questionnaire scores of children with mild asthma in the two groups at three months after the end of asthma education compared with those at the end of asthma education (VR group: *P* = 1.000; asthma education group: *P* = 0.073). However, both at the end of the intervention and three months after the end of the intervention, the C - ACT questionnaire scores of children with mild asthma in the VR group were higher than those in the asthma education group (after the end of the intervention: *P* < 0.001; three months after the end of the intervention: *P* < 0.001) (Table 3).

**Table 3:** Asthma control effects in children of different subgroups.

Parameter	Time	The VR group	The asthma education group	*t*	*P*
Children with severe asthma	At the end of intervention	20.20 ± 1.10	16.33 ± 0.58	5.547	0.001
	3 months after the end of intervention	21.80 ± 1.30	18.00 ± 1.00	4.297	0.005
Children with mild asthma	At the end of intervention	24.14 ± 1.61	20.61 ± 2.32	7.506	<0.001
	3 months after the end of intervention	24.14 ± 1.61	21.42 ± 1.50	7.474	<0.001

### 3.3 Emotional turmoil state of pediatric patients

Statistically speaking, prior to the intervention, the patients within the VR group exhibited a SCARED score of (65.55 ± 5.42) and a CDI score of (41.12 ± 5.01). In the asthma education group, the pre - intervention SCARED score stood at (66.02 ± 5.23) and the CDI score was (40.63 ± 5.20). There was no statistically significant disparity in the SCARED scores and CDI scores between the two cohorts of pediatric patients prior to the intervention (SCARED score: *P* = 0.690; CDI score: *P* = 0.666).

Subsequent to the intervention, the patients in the VR group registered a SCARED score of (31.45 ± 4.67) and a CDI score of (21.85 ± 4.54). In the asthma education group, the SCARED score was (49.41 ± 4.67) and the CDI score was (32.88 ± 4.23). The SCARED scores and CDI scores of both groups of pediatric patients diminished in comparison to those before the intervention (all *P* < 0.001), and the SCARED scores and CDI scores of pediatric patients in the VR group were lower than those in the asthma education group, with statistically significant distinctions (all *P* < 0.001) (Table 4).

**Table 4:** Emotional turmoil state of pediatric patients.

Parameter	Time	The VR group (*n* = 40)	The asthma education group (*n* = 41)	*t*	*P*
SCARED score	Before intervention	65.55 ± 5.42	66.02 ± 5.23	0.401	0.690
	After intervention	31.45 ± 4.67*	49.41 ± 4.67*	17.305	<0.001
CDI score	Before intervention	41.12 ± 5.01	40.63 ± 5.20	0.4328	0.666
	After intervention	21.85 ± 4.54*	32.88 ± 4.23*	11.315	<0.001

SCARED, screen for child anxiety related emotional disorders; CDI, children's depression inventory.

* Compared with the same group before intervention, *P* < 0.05.

### 3.4 Treatment adherence of children

Statistics indicated that the MMAS - 8 score of patients in the VR group was (6.40 ± 1.08). Among them, 8 children had an MMAS - 8 score below 6, signifying poor adherence, whereas 32 children had an MMAS - 8 score of 6 or above, suggesting better adherence. The MMAS - 8 score of patients in the asthma education group was (5.80 ± 1.15). There were 17 children with an MMAS - 8 score below 6, indicating poor adherence, and 24 children with an MMAS - 8 score of 6 or above, demonstrating better adherence. A comparison disclosed that the MMAS - 8 score of patients in the VR group was also higher than that in the health education group (*P* = 0.019). Furthermore, the number of patients with poor adherence during the intervention period in the VR group was fewer than that in the asthma education group (*P* = 0.037) (Table 5).

**Table 5:** Treatment adherence of children.

Parameter	The VR group (*n* = 40)	The asthma education group (*n* = 41)	*t*/X^2^	*P*
MMAS - 8 score	6.40 ± 1.08	5.80 ± 1.15	2.404	0.019
Number of children with poor compliance	8	17	4.371	0.037

MMAS-8, Morisky medication adherence scale.

### 3.5 Quality of life of the children

Statistically speaking, prior to the intervention, within the VR group, the score on the symptom dimension of the PAQLQ stood at (32.43 ± 4.90), the score on the activity limitation dimension of the PAQLQ was (19.20 ± 3.16), the score on the emotional function dimension of the PAQLQ reached (24.65 ± 3.34), and the overall PAQLQ score tallied up to (76.26 ± 5.93). In the asthma education group, before the intervention, the score on the symptom dimension of the PAQLQ was (30.29 ± 5.58), the score on the activity limitation dimension of the PAQLQ measured (18.56 ± 3.46), the score on the emotional function dimension of the PAQLQ amounted to (24.85 ± 2.91), and the total PAQLQ score equated to (73.71 ± 7.24). Upon making a comparison, there were no statistically significant disparities in the symptom, activity limitation, emotional function, and total scores of the PAQLQ between the two groups of children prior to the intervention (symptom dimension: *P* = 0.072; activity limitation dimension: *P* = 0.389; emotional function dimension: *P* = 0.771; total score: *P* = 0.085).

Following the intervention, in the VR group, the score on the symptom dimension of the PAQLQ climbed to (37.85 ± 3.44), the score on the activity limitation dimension of the PAQLQ rose to (24.25 ± 2.70), the score on the emotional function dimension of the PAQLQ ascended to (30.86 ± 4.50), and the overall PAQLQ score soared to (92.96 ± 6.51). In the asthma education group, the score on the symptom dimension of the PAQLQ reached (34.80 ± 5.06), the score on the activity limitation dimension of the PAQLQ attained (21.17 ± 3.75), the score on the emotional function dimension of the PAQLQ reached (27.46 ± 4.67), and the total PAQLQ score reached (83.44 ± 8.33). Upon comparison, the scores on the symptom, activity limitation, emotional function, and the total score of the PAQLQ in both groups of children were higher than those before the intervention (in the VR group: all *P* < 0.001; in the asthma education group: symptom dimension: *P* < 0.001; activity limitation dimension: *P* = 0.002; emotional function dimension: *P* = 0.003; total score: *P* < 0.001). Moreover, the scores on the symptom, activity limitation, emotional function, and the total score of the PAQLQ in the VR group were higher than those in the asthma education group, with statistically significant differences (symptom dimension: *P* = 0.002; activity limitation dimension: *P* < 0.001; emotional function dimension: *P* = 0.001; total score: *P* < 0.001).

Three months after the intervention ended, in the VR group, the score on the symptom dimension of the PAQLQ was (41.38 ± 5.17), the score on the activity limitation dimension was (24.25 ± 2.70), the score on the emotional function dimension was (30.86 ± 4.50), and the overall PAQLQ score was (92.96 ± 6.51). In the asthma education group, the score on the symptom dimension of the PAQLQ was (34.80 ± 5.06), the score on the activity limitation dimension was (21.17 ± 3.75), the score on the emotional function dimension was (27.46 ± 4.67), and the total PAQLQ score was (83.44 ± 8.33). Upon comparison, there were statistically significant differences in the symptom, activity limitation, emotional function, and total scores of the PAQLQ among the pre - intervention, post - intervention, and three - month - after - intervention time points in both groups of children (all *P* < 0.001). Moreover, the scores on the symptom, activity limitation, emotional function, and the total score of the PAQLQ at three months after the intervention ended were higher than those at the end of the intervention (VR group: symptom dimension: *P* < 0.001; activity limitation dimension: *P* = 0.006; emotional function dimension: *P* < 0.001; total score: *P* < 0.001; asthma education group: symptom dimension: *P* = 0.008; activity limitation dimension: *P* = 0.003; emotional function dimension: *P* < 0.001; total score: *P* < 0.001), and the scores in the VR group were higher than those in the asthma education group (symptom dimension: *P* < 0.001; activity limitation dimension: *P* = 0.001; emotional function dimension: *P* < 0.001; total score: *P* < 0.001) (Table 6).

**Table 6:** Quality of life of the children.

Parameter	Time	The VR group (*n* = 40)	The asthma education group (*n* = 41)	*t*	*P*
Score of the symptom dimension in PAQLQ	Before intervention	32.43 ± 4.90**	30.29 ± 5.58**	1.826	0.072
	After intervention	37.85 ± 3.44*	34.80 ± 5.06*	3.16	0.002
	3 months after the end of intervention	41.38 ± 5.17*^,^**	37.61 ± 4.18*^,^**	3.607	<0.001
F		38.982	22.586		
P		<0.001	<0.001		
Score of the activity limitation dimension in PAQLQ	Before intervention	19.20 ± 3.16**	18.56 ± 3.46**	0.867	0.389
	After intervention	24.25 ± 2.70*	21.17 ± 3.75*	4.230	<0.001
	3 months after the end of intervention	26.53 ± 4.29*^,^**	23.61 ± 3.52*^,^**	3.346	0.001
F		47.260	20.398		
P		<0.001	<0.001		
Score of the emotional function dimension in PAQLQ	Before intervention	24.65 ± 3.34**	24.85 ± 2.91**	0.293	0.771
	After intervention	30.86 ± 4.50*	27.46 ± 4.67*	3.346	0.001
	3 months after the end of intervention	46.55 ± 4.64*^,^**	37.44 ± 3.56*^,^**	9.932	<0.001
F		288.836	126.244		
P		<0.001	<0.001		
Total score of PAQLQ assessment	Before intervention	76.26 ± 5.93**	73.71 ± 7.24**	1.744	0.085
	After intervention	92.96 ± 6.51*	83.44 ± 8.33*	5.728	<0.001
	3 months after the end of intervention	114.45 ± 13.92*^,^**	98.66 ± 11.14*^,^**	5.645	<0.001
F		161.957	79.109		
P		<0.001	<0.001		

PAQLQ, pediatric asthma quality of life questionnaire.

* Compared with the same group before intervention, *P* < 0.05.

** Compared with the post - intervention data within the same group, *P* < 0.05.

### 3.6 Assistance - seeking situations of the children and their families

Statistically, in the VR group, the average daily number of times the children and their families accessed educational materials through the VR device was (10.53 ± 3.67), and there were no cases of seeking other forms of help such as via email or phone in this group. In the asthma education group, the average daily number of times the children and their families sought additional help was (3.54 ± 1.14). Upon comparison, the average daily number of times the children in the VR group accessed educational materials was significantly higher than that of the children in the asthma education group (*P* < 0.001).

### 3.7 Satisfaction with VR

After interviewing and conducting statistics on the parents of the patients, 37 (92.5%) parents of the children reported being satisfied with the VR device used and the asthma education based on the VR device. They stated that the VR device and the corresponding training and education improved the children's understanding of the disease, reduced the number of acute attacks, and enhanced the children's self - care ability. Meanwhile, 15% of the parents thought that their children had difficulties operating the VR device independently.

## 4 Discussion

Asthma education, as an educational initiative directed at asthma patients and their caregivers, can efficaciously enhance patients' asthma management outcomes, fortify patients' confidence in the treatment, augment medication adherence, and cultivate a favorable rapport with physicians (31). For children afflicted with bronchial asthma, traditional asthma education fails to possess sufficient allure, impeding children from receiving efficacious and continuous asthma education, which, in turn, undermines the interventional efficacy of asthma education (32). This study, through the integration of VR technology with asthma education, reveals that VR - based asthma education effectively bolsters the asthma control of children with bronchial asthma, alleviates their levels of anxiety and depression, enhances their treatment compliance, and elevates their quality of life.

Children with bronchial asthma are more susceptible to negative emotions such as anxiety and depression. Once these negative emotions manifest, the asthma control efficacy may wane (33). On one hand, when confronted with bronchial asthma, due to inadequate comprehension of the disease and an increase in the perceived number of stressors that are arduous to handle, children bear excessive psychological burdens. This, in turn, affects their physiological functions, leading to a down - regulation of effector receptors, the establishment of a pro - inflammatory milieu with a hypo - reactive hypothalamic - pituitary - adrenal axis in the body, and a diminished response to therapies such as short - acting bronchodilators, thus resulting in suboptimal asthma control (34). On the other hand, since bronchial asthma is a malady necessitating long - term treatment, children mostly passively fulfill certain tasks to adhere to medical advice during treatment. This diminishes their sense of enjoyment in treatment, resulting in poor treatment compliance and ultimately subpar asthma control (35).

In this study, we discovered that after children received the intervention integrating VR technology with asthma education, their asthma control, negative emotions, and treatment compliance improved to a greater degree compared with those who received conventional asthma education. This indicates that VR - based asthma education plays a pivotal role in ameliorating children's asthma control status, negative emotions, and treatment compliance. This may be because the integration of VR technology in the asthma education process endows it with a sense of immersion and interactivity. Moreover, VR technology enables children to remain engrossed in the content during asthma education, evading interference from the surrounding environment and further enhancing the efficacy of asthma education. Through VR technology, children acquire a clearer understanding of asthma - related knowledge and the utilization of asthma medications, alleviating the stress they encounter when dealing with bronchial asthma (36–38). Meanwhile, when children utilize VR devices, the diversity and allure of VR resources arouse their greater interest in treatment. In this process, the increased communication between children, parents, and medical staff allows children to maintain a high level of enjoyment in treatment, which also contributes to the improvement of their emotional state (39, 40).

Asthma education, as an indispensable component of clinical asthma treatment, ultimately aims to enhance the quality of life of asthma patients (41). This study demonstrates that children who received VR - based asthma education enjoy a superior quality of life compared with those who received conventional asthma education, indicating the role of VR - based asthma education in enhancing the quality of life of children with bronchial asthma. The reason may be that after acquiring more knowledge about bronchial asthma, children adopt a positive outlook towards asthma treatment. They can effectively recognize the symptoms of bronchial asthma and take proactive and efficacious countermeasures, which mitigates the severity of asthma attacks, reduces the impact of bronchial asthma on their lives and studies, and thus instills confidence in life, heightens their sense of well - being, and ultimately elevates their quality of life (42, 43).

In addition, this study found that children with asthma and their families using VR devices significantly reduced the frequency of seeking other forms of help by accessing educational materials. This helps to reduce the workload of the staff. Meanwhile, the reduced need for additional help in the VR group also reflects the advantages of self - paced and repeated learning (44). Moreover, 92.5% of the children's families were satisfied with the devices and the asthma education methods used in this study, which indicates a high level of satisfaction with VR - based asthma education. However, at the same time, some families were concerned about the operational complexity of VR devices. This shows that although VR technology has high acceptability, for children with limited access to technology, auxiliary training by nursing staff may be necessary. These insights are consistent with previous studies, emphasizing the role of user - centered design in pediatric VR applications (45).

Undoubtedly, this study has certain limitations. Firstly, the sample size in this study was relatively small, and most of the study subjects were from urban communities and visited a single hospital. This restricted the source of the study sample. Meanwhile, this study did not evaluate the social, cultural, economic, and educational backgrounds of the children. Children with bronchial asthma from rural areas or those with low education/low - income families often have a poorer quality of life due to various reasons. This may limit the application of this study on a large scale, in multi - centers, and in rural communities. Additionally, the intervention period for children in this study is relatively brief. The long - term impact of VR - based asthma education on the quality of life of children with bronchial asthma still necessitates longer - term intervention and observation in subsequent studies.

In conclusion, VR - based asthma education effectively enhances the asthma control of children with bronchial asthma, alleviates their levels of anxiety and depression, improves their treatment compliance, and ultimately elevates their quality of life. Therefore, the integration of VR technology with asthma education can be further investigated and promoted in clinical and non - clinical settings to improve the current state of asthma education for children with bronchial asthma and foster their healthy development.

## Data Availability

The original contributions presented in the study are included in the article/Supplementary Material, further inquiries can be directed to the corresponding author.
